# Delivering diabetes care in underserved communities in South Africa: Insights from the 2025 Diabetes Summit Workshop

**DOI:** 10.4102/phcfm.v18i2.5579

**Published:** 2026-08-18

**Authors:** Khutso G. Phalane, Wyvine A. Bapolisi, Nelisiwe Mkize, Rivoningo Gonani, Kibachio J. Mwangi, Melanie Pienaar, Patrick Ngassa Piotie

**Affiliations:** 1 Health and Related Sciences, Science Advisory Programme, Academy of Science of South Africa, Pretoria, South Africa; 2 Center for Tropical Diseases and Global Health, School of Medicine, Catholic University of Bukavu, Bukavu, Democratic Republic of the Congo; 3 Department of Internal Medicine, School of Clinical Medicine, Faculty of Health Sciences, University of the Witwatersrand, Johannesburg, South Africa; 4 Academy of Science of South Africa, Pretoria, South Africa; 5 World Health Organization, Pretoria, South Africa; 6 School of Nursing, Faculty of Health Sciences, University of the Free State, Bloemfontein, South Africa; 7 School of Health Systems and Public Health, Faculty of Health Sciences, University of Pretoria, Pretoria, South Africa; 8 Department of Public Health, Medical School, Nelson Mandela University, Gqeberha, South Africa

## Introduction

Diabetes prevalence is rising rapidly in South Africa,^1,2^ placing increasing pressure on the health system and disproportionately affecting underserved populations in rural, peri-urban and informal settlement contexts.^3^ These communities face persistent structural barriers, including geographic isolation, workforce shortages, fragmented referral pathways and frequent stockouts of essential medicines and diagnostic tools.^4,5^ In addition, adverse social determinants of health further compromise diabetes care, particularly low socio-economic conditions such as food insecurity, housing instability and financial barriers to accessing healthcare services and medications.^6^ In response to these challenges, a dedicated workshop titled *Reaching the Unreached: Delivering Diabetes Care in Underserved Communities* was convened during the 2025 Diabetes Summit.^7^ Organised by the Diabetes Alliance in collaboration with partners, including the Academy of Science of South Africa (ASSAf), Rural Doctors Association of Southern Africa (RuDASA), the Tshemba Foundation, Doctors Without Borders (MSF), and the Non-Communicable Diseases and Injuries (NCDI) Poverty Network, the workshop brought together clinicians, policymakers, community leaders, researchers and people living with diabetes to share experiences and co-develop solutions. The workshop aimed to move beyond problem identification towards practical, scalable strategies for strengthening equitable diabetes care. This article synthesises key insights, innovations and recommendations emerging from the session, drawing on a comprehensive report of the workshop proceedings.

## Understanding the reality: Lived experience at the centre

A defining feature of the workshop was the prioritisation of lived experience as a source of evidence. Individuals living with diabetes face persistent challenges, including stigma, lack of awareness and limited access to essential supplies such as insulin, glucometers and test strips. Participants described how misconceptions, such as conflating type 1 and type 2 diabetes, continue to shape community attitudes and delay care-seeking. Experiences shared during the session illustrated that, in some communities, a diabetes diagnosis is still perceived as a ‘death sentence’, reflecting deep gaps in education and psychosocial support for individuals living with diabetes. Importantly, people living with diabetes were not positioned as passive recipients of care but as active agents of change. Examples include community-led screening initiatives, peer support groups and advocacy efforts aimed at reducing stigma and improving awareness of diabetes. These contributions reinforce the importance of embedding lived experience into programme design, implementation and evaluation.

## Frontline realities: Delivering care in resource-constrained settings

Frontline healthcare workers provided critical insights into the practical challenges of delivering diabetes care in underserved settings. Case-based presentations highlighted systemic gaps, such as delayed diagnosis, poor continuity of care and limited access to multidisciplinary services. Innovative responses have emerged despite these constraints. For example, integrated, patient-centred care models implemented in rural hospitals have demonstrated improvements in outcomes through multidisciplinary teamwork, group education and community engagement. These models often extend beyond clinical care to include social support, linkage to social protection services and collaboration with community health workers and traditional leaders in the community. However, the persistent stockouts of insulin, syringes and diagnostic tools remain a major barrier. Participants emphasised that innovation cannot compensate for the absence of basic resources, highlighting the ethical and clinical challenges of managing chronic conditions without essential supplies.

## Harnessing technology and innovation to expand access in underserved communities

The workshop underscored the need for a broad understanding of innovation in diabetes care. While digital health tools and artificial intelligence (AI) offer new opportunities, many impactful innovations are low-cost, community-driven and contextually adapted to the local environment.

Technology-enabled solutions presented during the session included electronic health records, AI-supported clinical decision tools and point-of-care diagnostics that enable decentralised care delivery. These innovations can improve access to specialist input, reduce unnecessary referrals and support clinical decision-making in resource-limited settings. Simultaneously, community-based innovations have emerged as critical enablers of access to diabetes care in underserved settings. Decentralised medication pick-up points reduce geographic and financial barriers, thereby improving treatment adherence and continuity of care. Community health worker-led outreach and monitoring strengthened linkage to care and supported ongoing patient engagement. Peer support groups and diabetes camps address important psychosocial dimensions of diabetes management, including stigma, isolation and self-efficacy. Locally driven initiatives, often supported by traditional leadership structures, further illustrate the importance of community ownership and contextually grounded approaches to improve access to care and health outcomes. A key message emerging from these examples is that innovation must be people-centred, embedded within existing health systems and responsive to local realities.

## Community-based models of care: Lessons for scale-up

Several scalable models of care were discussed, offering practical pathways for strengthening diabetes services in underserved areas. The PEN-Plus model,^8^ implemented across multiple African countries, demonstrates how task-shifting to nurses and clinical officers can expand access to care for severe non-communicable diseases (NCDs) at the district level. Supported by structured mentorship and peer support, this model has shown that high-quality diabetes care can be delivered outside tertiary centres. Similarly, community-based medication distribution models implemented by Doctors Without Borders in South Africa illustrate how decentralisation can improve continuity of care. By bringing services closer to communities, these models reduce financial and logistical barriers while strengthening patient involvement. Several common enabling factors emerged from these examples. Strong community engagement and local ownership were central to the success and sustainability of these interventions. The integration of psychosocial support into care models enhances patient outcomes by addressing broader determinants of health. Continuous training and mentorship of frontline healthcare workers strengthened service delivery capacity, while collaboration between government, non-governmental organisations and communities facilitated coordinated and scalable responses. Collectively, these models underscore the importance of shifting care closer to communities while maintaining robust referral systems and appropriate clinical oversight.

## Health system challenges: Policy, financing and accountability

The discussions during the panel session highlighted the structural challenges that limit the scale-up of effective models. Although policy frameworks such as the National Strategic Plan for NCDs provide a foundation,^9^ gaps in implementation persist, particularly in workforce capacity, supply chain management and data systems. Participants emphasised the need for stronger accountability mechanisms to address persistent stockouts and ensure equitable access to essential medicines and diagnostics. The role of systems such as the Stock Visibility System was acknowledged, but variability in performance across provinces remained a concern for the participants. Financing was identified as a critical barrier, with participants noting the historical underfunding of NCDs compared to communicable diseases. Calls were made for increased investment and stronger engagement with the National Treasury to secure sustainable funding for diabetes care. Importantly, the workshop highlighted the need for multisectoral collaboration. Addressing diabetes effectively requires coordinated action across health, education, agriculture and social development sectors to tackle underlying determinants such as food insecurity and poverty.

## Key recommendations for advancing diabetes care

The workshop generated a set of actionable recommendations aimed at strengthening diabetes care in underserved communities (Table 1).

**TABLE 1:** Key recommendations for strengthening diabetes care in underserved communities.

Priority area	Recommendation
Formalising the role of people living with diabetes	Integrate lived experience into policy, programme design and service delivery, with appropriate training and support for peer educators and advocates.
Scaling decentralised and task-shared models of care	Expand community-based and district-level services, supported by mentorship and referral systems to reduce reliance on tertiary care.
Ensuring reliable access to essential resources	Prioritise an uninterrupted supply of medicines, diagnostics and monitoring tools, with strengthened supply chain accountability.
Integrating psychosocial support	Embed mental health services, peer support and social protection mechanisms into routine diabetes care.
Strengthening governance and financing	Promote whole-of-government approaches and mobilise sustainable funding to address the systemic underinvestment in noncommunicable diseases.

These recommendations reflect a shift towards people-centred, equitable and system-oriented approaches to diabetes care.

## Conclusion

The *Reaching the Unreached* workshop highlighted both the depth of the challenges and the breadth of the solutions emerging across South Africa and beyond. Underserved communities face significant barriers to diabetes care, but they are also sites of innovation, resilience and leadership. A central lesson from the workshop was that improving diabetes outcomes requires more than technological innovation. This demands systemic change grounded in equity, community engagement and accountability. Scaling effective models will require sustained investment, stronger partnerships and a deliberate commitment to placing people living with diabetes at the centre of care. Ultimately, reaching the unreached is not only a technical challenge but also a moral imperative. Ensuring equitable access to diabetes care is essential for achieving the broader goals of health system strengthening and social justice in South Africa.
